# Deployment-Based Lifetime Optimization Model for Homogeneous Wireless Sensor Network under Retransmission

**DOI:** 10.3390/s141223697

**Published:** 2014-12-10

**Authors:** Ruiying Li, Xiaoxi Liu, Wei Xie, Ning Huang

**Affiliations:** 1 School of Reliability and Systems Engineering, Beihang University, No.37 Xueyuan Road, Haidian District, Beijing 100191, China; E-Mails: nora0429@163.com (X.L.); hn@buaa.edu.cn (N.H.); 2 Science and Technology on Reliability and Environmental Engineering Laboratory, No.37 Xueyuan Road, Haidian District, Beijing 100191, China; 3 China CEPREI Laboratary, Guangzhou 510610, China; 4 Department of Systems and Industrial Engineering, University of Arizona, Tucson, AZ 85721, USA; E-Mail: wxie@email.arizona.edu

**Keywords:** wireless sensor networks, sensor deployment, lifetime optimization, retransmission, energy consumption

## Abstract

Sensor-deployment-based lifetime optimization is one of the most effective methods used to prolong the lifetime of Wireless Sensor Network (WSN) by reducing the distance-sensitive energy consumption. In this paper, data retransmission, a major consumption factor that is usually neglected in the previous work, is considered. For a homogeneous WSN, monitoring a circular target area with a centered base station, a sensor deployment model based on regular hexagonal grids is analyzed. To maximize the WSN lifetime, optimization models for both uniform and non-uniform deployment schemes are proposed by constraining on coverage, connectivity and success transmission rate. Based on the data transmission analysis in a data gathering cycle, the WSN lifetime in the model can be obtained through quantifying the energy consumption at each sensor location. The results of case studies show that it is meaningful to consider data retransmission in the lifetime optimization. In particular, our investigations indicate that, with the same lifetime requirement, the number of sensors needed in a non-uniform topology is much less than that in a uniform one. Finally, compared with a random scheme, simulation results further verify the advantage of our deployment model.

## 1. Introduction

Due to the technological developments of microelectromechanical systems, wireless communication and digital electronics, it becomes possible to produce low-cost wireless sensors. A wireless sensor network (WSN), which consists of a number of sensor nodes (several tens to thousands working together), is applied for information monitoring in the fields such as health monitoring [1], military target tracking and surveillance [2], natural disaster relief, hazardous environment exploration [3], intelligent transportation [4], and social networking and gaming [5].

Because the sensors have limited energy resource, the lifetime of a WSN is significantly affected by its energy consumption [6]. Thus, improving the energy efficiency becomes a hot topic in this area [7,8]. Anastasi *et al.* [9] state that the energy consumed by a WSN is mainly used for communication and data processing. Evidently, the energy consumed by communication is sensitive to the transmission distance. Thus, sensor deployment optimization is one of the most important methods used to reduce the energy consumption [7,10]. In many applications, the data gathered via WSN are essential since the transmission errors may lead to system failures that may cause economic losses, environmental damage or casualties [11]. As WSN is usually deployed in a harsh environment, according to the reports, the packet loss ratio can be as high as 70% in a typical WSN [12]. Retransmission is now widely applied to improve the success rate of data transmission [13–15]. It is obvious that data retransmission consumes extra energy. However, previous work usually assumes that the data transmission is always successful and neglects the possible retransmission.

In this paper, the lifetime of a WSN is defined as the period starting from the initial working time until the WSN fails to satisfy its requirements (including coverage, connectivity and success transmission rate). Our goal is to maximize the lifetime for a homogeneous WSN that is used to monitor a circular target area with a base station in the center. We propose a new sensor deployment optimization model based on the energy consumption calculated under retransmission. The remainder of this paper is organized as follows. In Section 2, we briefly review the related work of sensor deployment optimization. Section 3 describes the static topology and dynamic behaviors of the WSN in our problem. The lifetime optimization models are proposed for both uniform and non-uniform deployment problems in Section 4. We analyze the energy consumption in Section 5, while the path probability and the success rate of data transmission, with and without retransmission, are quantified in Section 6, respectively. In Section 7, two case studies are presented to verify the effectiveness of considering retransmission in the lifetime optimization, and the advantage of non-uniform deployment scheme compared with a uniform one. Simulation further demonstrates that our optimal deployment scheme can provide much longer lifetime and higher successful transmission rate than a random scheme. Finally, Section 8 concludes our results.

## 2. Related Work

There are two types of sensor deployment schemes: structured and unstructured. The difference between these two schemes is that the sensors of an unstructured WSN are randomly deployed (this is usually used in an unreachable environment) while the positions of the sensors in a structured one are pre-determined. In this paper, we focus on the structured scheme.

The structured sensor deployment problem has been extensively studied by many researchers, and pioneers deploy such pre-determined WSN in practice, such as WSNs used to monitor Dolmabahçe Palace (Istanbul), Torre Aquila (Italy), a local street (Spain), Bird's Nest (China) and a forested nature reserve (USA), see Onur *et al.* [16], Ceriotti *et al.* [17], Gallart *et al.* [18], Shen *et al.* [19] and Navarro *et al.* [20], respectively. Onur *et al.* [16] claim that deterministic deployment is appropriate for plain and easily accessible fields such as the embassy/museum garden. For this type of deployment, the number of sensors and the topology of WSN are determined in advance. Thus, the associated energy consumption and lifetime of the WSN can be analyzed and optimized before the deployment. Wu *et al.* [8], Liu *et al.* [21] and Wang [22] divide the target area into multiple rings and analyze the optimal sensor deployment schemes in different rings. AbdelSalam [23], Chiang *et al.* [24], Fan *et al.* [25] and Gupta *et al.* [26] study the structured deployment methods based on grids, and Onur *et al.* [16] deployed a WSN based on grids to monitor Dolmabahçe Palace in Istanbul. In [27], the advantages and potential applications of hexagon-based WSN are introduced, and the study shows that WSN can gain benefits from the hexagon-based topology in coverage, energy saving, reliability, routing design, *etc.* The deployment of such hexagon-based topology requires to determine the sensor locations according to the coordinate. For those key WSNs with long lifetime requirement, such as the WSN that may be used in the cabin of the next generation airborne networks (see Yedavalli and Belapurkar [28], Leipold *et al.* [29] and Wang and Hu [30]), it is worthy to deploy sensors according to the optimal deployment scheme.

The WSN topologies can be either heterogeneous or homogeneous. Compared with the heterogeneous WSN, the homogeneous one, in which all sensors are the same in sensing capability, communication capability, power capability, *etc.*, is convenient and cheap to deploy. Pioneers propose several sensor deployment schemes for homogeneous WSN. For instance, Wang [22] applies the hexagonal grids to investigate the minimal sensor density. He assumes that the whole target area is a circular plane that can be divided into rings with a centered base station. The relationship between the sensor density and the ring radius is studied. However, the exact positions of these sensors are not given, which leads to a large overlap of coverage or no coverage in some areas. Gupta *et al.* [26] divide the target area into regular hexagonal grids, in which sensors are deployed in the middle of the grids. They indicate that the deployment can achieve the optimal coverage and, at the same time, satisfy the connectivity constraints, but the energy consumption and the success transmission rate of WSN are not considered. Tian *et al.* [27] also study the deployment based on the regular hexagonal grids. They address the advantages of their method in coverage, connectivity, reliability and energy consumption, but, to achieve the optimal lifetime and sensor deployment scheme, the distance between the neighboring sensors and the number of sensors deployed are not optimized. Moreover, retransmission, which is an important way to guarantee the success data transmission rate, is ignored in all of the abovementioned research works.

In this paper, retransmission, which has a large effect on the energy consumption but is usually neglected in previous works, is taken into account in our deployment-based lifetime optimization. A regular hexagonal grid deployment scheme, which can achieve the optimal coverage and connectivity as mentioned earlier, is presented as our basic deployment scheme. Based on the data transmission and energy consumption analysis under retransmission, the optimal sensor deployment is obtained by maximizing the lifetime under the constraints of coverage, connectivity and data success transmission rate. As the retransmission rate is highly related to the transmission distance, the optimal sensor deployment under retransmission is quite different from the one in which retransmission is not considered. Moreover, our scheme can guarantee the coverage, connectivity and data success transmission rate, which may not be achieved simultaneously in previous works.

## 3. Problem Description

In this paper, we assume that the target monitoring area is circular with a radius of *R_a_*, and its base station lies in the center (the same problem can be seen in [8,22,23,31], *etc.*). In this section, the static topology of a WSN is presented for coverage and connectivity analysis, and its dynamic behaviors are described for further discussion.

### 3.1. Static Topology

**Figure 1.**
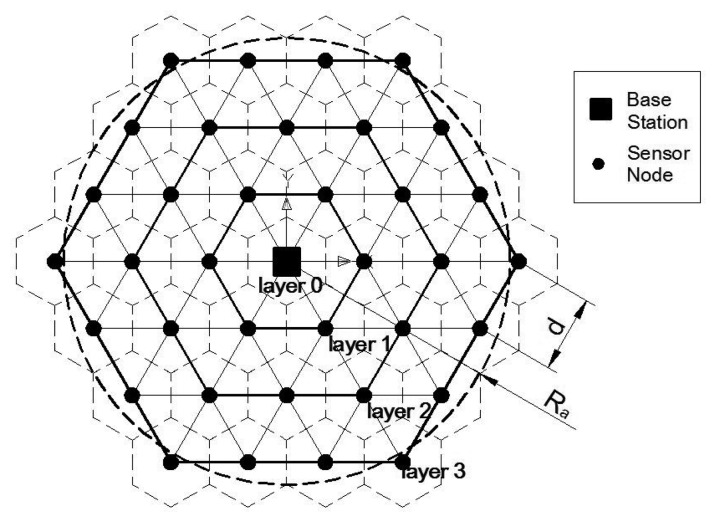
The topology of homogeneous WSN.

We discuss the sensor deployment scheme based on a regular hexagonal topology, because the minimal overlap of coverage can be achieved by partitioning the target monitoring area into hexagonal grids, in which the sensors are centered, without overlap of physical area (see [27]). As shown in Figure 1, a homogeneous WSN, made up by many identical wireless sensors and a base station, is used to monitor the target area. The base station is located in the center of the circular target area, and one or more sensors are deployed in the middle of each hexagonal grid around the base station. The Mica2 mote, the most commonly used sensor [32], is used in this homogeneous WSN. Its parameters can be found in the CC1000 datasheet [33]. The sensors have the same initial energy *E*_0_, while the initial energy of base station is unlimited. Each sensor has a sensing radius of *R_s_* and a transmission radius of *R_t_*. Figure 1 depicts the geometric formulation of these regular hexagonal grids leads to multiple layers (the layer number increases from inside to outside), where the base station is located in Layer 0. The distance between the neighboring sensors is denoted *d*, so the circumcircle radius of each regular hexagonal grid can be expressed as $\frac{\sqrt{3}d}{3}$, and the circumcircle radius of a sensor in Layer *i* can be calculated as *d_c_*(*i*) = *id*.

#### 3.1.1. Coverage Analysis

As shown in Figure 1, to satisfy the requirement of covering the entire target area, the distance between neighboring sensors should satisfy

(1) $$d\leq \sqrt{3}R_{s}.$$

The overlap of coverage of the network is minimized at $d=\sqrt{3}R_{s}$. We know that the overlap of coverage increases as the value of *d* decreases, which results in energy wastage. On the other hand, the larger *d* is, the more energy will be consumed to transmit a message in a single hop. This implies the necessity of choosing an optimal value for *d*.

#### 3.1.2. Connectivity Analysis

With these regular hexagonal grids, if the whole target area is covered, when

(2) $$d\leq R_{t},$$

the sensors in the region are connected.

### 3.2. Dynamic Behavior

**Figure 2.**
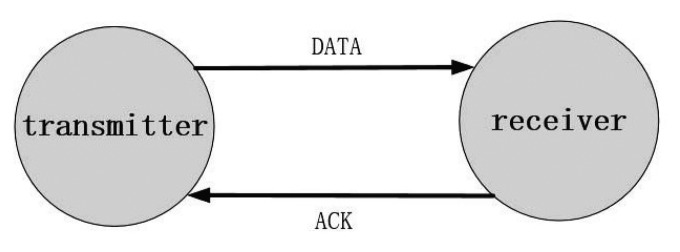
Reduced CSMA/CA protocol without RTS/CTS.

Besides the transmission distance, the energy consumption is also affected by the network protocols. In each data gathering cycle, sensors can detect the environment information within its sensing range and transmit *a* packets, each with *m_data_* bits, to the base station hop-by-hop. Generally, the sensor will not fail unless its energy is exhausted. In this paper, we study the sensor deployment optimization problems based on the following protocol assumptions:

- Routing protocol. The Greedy Perimeter Stateless Routing (GPSR) algorithm is applied to determine the next hop for transmitting data. In GPSR, it is preferred to forward the packet of a sensor to the neighboring sensor, which is the closest one to the base station, within its transmission range [34]. To ensure that the data can only be transmitted between the sensors of neighboring grids, we assume
  (3) $$d>\frac{\sqrt{3}R_{t}}{3}.$$
- Sleep/wake-up protocol. Since a single sensor will run out of energy easily, it is common to deploy some redundant units. The Probing Environment and Adaptive Sleeping (PEAS) protocol proposed by Ye *et al.* [35] is adopted in our model to control the sleep/wake-up mechanism. The protocol wakes a sensor up only when no sensor is active within its probing range (which is usually the same as the sensing range). Thus, we assume that
  (4) $$d>R_{s}$$
  It is reasonable that much overlap exists and energy is wasted if *d* ≤ *R_s_*. According to this protocol, at each sensor-deployed location, *i.e.*, the center of each hexagonal grid in Figure 1, only one sensor is active that can be used to detect, transmit and receive message. The other sensors are in sleeping status with little energy consumption (ignorable).
- Retransmission protocol. Retransmission is widely used to improve the success rate of data transmission. In this paper, the reduced CSMA/CA protocol without RTS/CTS is adopted. Figure 2 illustrates the data transmission process. The transmitter sends DATA message to its next hop according to the routing protocol. If the receiver receives the message successfully, an ACK message will be sent back to the transmitter. Note that, the transmitter will keep on sending DATA message repeatedly until it receives the ACK message or reaches the maximum number of retry attempts. In our case, the maximum number of retry attempts is assumed to be “2”.

As suggested by Zuniga *et al.* [36], the retransmission rate can be measured by packet reception rate. For a Mica2 mote using non-coherent frequency-shift keying modulation and encoded by Manchester, according to the IEEE 802.15.4 standard [37], its retransmission rate can be expressed as follows:

(5) $$RR\left(d\right)=\{\begin{array}{ll} 1-{\left[1-\frac{1}{2}e^{-\left(\frac{P_{t}-20\log_{10}\left(\frac{4\pi fd}{C}\right)-S_{r}}{1.28}\right)}\right]}^{2m-\text{l}}, & \text{if}\;d\leq 8\text{m},\\ 1-{\left[1-\frac{1}{2}e^{-\left(\frac{P_{t}-20\log_{10}\left(\frac{32\pi f}{C}\right)-33\log_{10}\left(\frac{d}{8}\right)-S_{r}}{1.28}\right)}\right]}^{2m-\text{l}}, & \text{otherwise}, \end{array}$$

where *P_t_* is the transmission power, *f* is the band width, *C* is the speed of light, *d* is the transmission distance, *S_r_* is the receiver's sensitivity, and *m* and *l* are the lengths of a single packet and the preamble (in bits), respectively.

## 4. Optimization Model

In this section, the lifetime optimization models are proposed. As Dietrich and Dressler stated in [38], the lifetime of the WSN is largely determined by energy depletion. Therefore, the energy consumption at each sensor location in a data gathering cycle is applied to calculate the lifetime of the network. Our goal is to cover the entire target area with full connectivity, and the WSN life ends when it cannot provide such coverage or connection function. By combining Equations (1) and (4), to ensure the coverage of a WSN, at least one sensor should be working in each hexagonal grid. To prolong the lifetime, as mentioned in Section 3, *n_i_*_,_*_j_* sensors are deployed in grid (*i, j*), among which only one is active and the rest are in sleeping mode due to the sleep/wake-up protocol. The lifetime of the WSN can be calculated by

(6) $$\tau =\overset{L}{\underset{i=1}{\min}}\left[\overset{6i}{\underset{j=1}{\min}}\left(\frac{n_{i,j}E_{0}}{E_{i,j}}\right)\right]t,$$

where *E*_0_ is the initial energy of a sensor, *E_i_*_,_*_j_* is the energy consumption in a data gathering cycle at location (*i, j*), *t* is the data gathering cycle and *L* is the number of layers in the target area. If the distance between sensors, *d*, is too small, the overlap of coverage will be very large, which results in huge amounts of redundant sensing information and energy waste. On the other hand, an extremely large value of *d* will dramatically increase the transmission energy of a single hop (since the transmission distance is increased, see Equation (12)). From all of the aforementioned perspectives, our objective is to achieve the optimal *d* and *n_i_*_,_*_j_* as well as maximize the WSN lifetime (which ends when the coverage, the connectivity or the success transmission rate falls below the designated threshold).

Here, we analyze two types of sensor deployments, uniform and non-uniform, which are usually discussed and compared in the deployment-based lifetime optimization. In a uniformly deployed WSN, the sensor density will not be changed by its distance to the base station. Such a deployment is simple, but it may cause energy hole problem. Thus, non-uniform deployment scheme was proposed (see [9]), in which more sensors are deployed near the base station, while less ones are located at the edge.

### 4.1. Uniform Deployment Optimization

For a uniform deployment scheme, the value of *n_i_*_,_*_j_* is a constant. Full coverage of the target area is required before the deployment is initialized. If all sensors in some grid of the network are out of energy, the coverage requirement will not be satisfied. The problem of finding an optimal *d* that maximizes the WSN's lifetime where only one sensor is located at each grid, subject to the coverage, connectivity and success transmission rate constraints, can be formulated as

(7) $$\begin{array}{rr} \max & \overset{L}{\underset{i=1}{\min}}\left(\overset{6i}{\underset{j=1}{\min}}\frac{E_{0}}{E_{i,j}}\right)t\\ \text{subject to} & S>S*,\\ & R_{s}<d\leq \sqrt{3}R_{s},\\ & \frac{\sqrt{3}}{3}R_{t}<d\leq R_{t}, \end{array}$$

where S* is the requirement of the success transmission rate. In Equation (7) *S* > S* requires that the success transmission rate for the whole network is at least S*, *d* > *R_s_* is the assumption for PEAS (see Equation (4)), $d\leq \sqrt{3}R_{s}$ is the requirement of the full coverage for the target area (see Equation (1)), $d>\frac{\sqrt{3}}{3}R_{t}$ is the assumption that data transmission is constrained between neighboring sensors (see Equation (3)), and *d* ≤ *R_t_* is the requirement of the connectivity between the locations of neighboring sensors (see Equation (2)).

This model is a nonlinear programming problem, which can be solved by the generalized reduced gradient method. If the lifetime requirement of the WSN is specified as τ*, the minimal number of sensors at location (*i,j*) can be calculated as

(8) $$n_{i,j}=\left\lceil \frac{\tau *}{\tau }\right\rceil ,$$

where *τ* is the optimal lifetime. The minimal total number of sensors is

(9) $$N=3L\left(L+1\right)\;\left\lceil \frac{\tau *}{\tau }\right\rceil ,$$

where *L* is the number of layers.

### 4.2. Non-Uniform Deployment Optimization Model

In the uniformly distributed homogenous WSN, sensors that are close to the sink consume energy faster than those far away from the base station due to the unevenly distributed forwarding workloads among sensors. Olariu and Stojmenović [39] have proved that the uneven energy depletion phenomenon is intrinsic to the system and no routing strategy can avoid the creation of an energy hole around the sink. However, the uneven energy depletion can be prevented by judicious system design, which can result in balanced energy consumption in the network. In a non-uniform deployment, *n_i_*_,_*_j_* is a variable based on different grids. The problem of finding the optimal *d* and *n_i_*_,_*_j_* that maximize the WSN's lifetime subject to coverage, connectivity, success transmission rate and number of sensors can be expressed as

(10) $$\begin{array}{rr} \max & \overset{L}{\underset{i=1}{\min}}\left(\overset{6i}{\underset{j=1}{\min}}\left(\frac{n_{i,j}E_{0}}{E_{i,j}}\right)\right)t\\ \text{subject to} & S>S*,\\ & R_{s}<d\leq \sqrt{3}R_{s},\\ & \frac{\sqrt{3}}{3}R_{t}<d\leq R_{t},\\ & \overset{L}{\underset{i=1}{\text{∑}}}\left(\overset{6i}{\underset{j=1}{\text{∑}}}n_{i,j}\right)\leq N^{*}, \end{array}$$

where N* is the maximum number of sensors. The new constraint $\overset{L}{\underset{i=1}{\text{∑}}}\left(\overset{6i}{\underset{j=1}{\text{∑}}}n_{i,j}\right)\leq N^{*}$ is a requirement that the number of sensors cannot exceed the maximum allowable number. For uniform deployment optimization problem, the lifetime of the WSN is proportional to the number of sensors in the sensor location. Hence, once the optimal sensor deployment scheme is determined for a WSN with only one sensor at each sensor location, it is the optimal deployment scheme for cases with more sensors at one location. Unlike the uniform deployment optimization problem discussed in Section 4.1, the number of sensors in a sensor location is not limited to 1. If no maximum allowable number of sensors is specified, the optimization objective, *i.e.*, the WSN lifetime, steadily increases with rising number of sensors in the network.

A genetic algorithm can be applied to solve this nonlinear integer programming problem. If the lifetime requirement of the WSN is specified as τ*, the minimal number of sensors can be obtained by

(11) $$N=\sum_{i=1}^{L}\left(\sum_{j=1}^{6i}\left\lceil \frac{\tau^{*}E_{i,j}^{\prime }}{tE_{0}}\right\rceil \right),$$

where $E_{i,j}^{\prime }$ is the optimal energy consumed by the sensor at location (*i,j*) in a data gathering cycle.

## 5. Energy Consumption

To determine the WSN lifetime in the optimization models, the energy consumption at each sensor location should be calculated. Generally, three statuses, including transmitting, receiving, and idle, of a sensor will cause energy consumption (see [40,41]). One widely used energy model presented in [42] is adopted in our problem. The basic energy consumption models for transmitting, receiving, and idle can be expressed as

(12) $$\{\begin{array}{c} Et=\left(\beta_{1}+\beta_{2}d^{\alpha }\right)m_{t},\\ E_{r}=\beta_{3}m_{r},\\ E_{id}=\beta_{4}t_{id}Pm_{\text{data}}, \end{array}$$

where *β*_1_ depends on the amount of energy spent in the electronics circuitry for transmitting each bit data, *β*_2_ is affected by the transmit amplifier efficiency, antenna gains and other system parameters, *β*_3_ is the amount of energy spent in the electronics circuitry for receiving each bit data, and *β*_4_ is the energy spent for each bit message in the idle state. Here *α* represents the path loss exponent (2 ≤ *α* ≤ 4), which depends on the environment. The parameters *m_t_, m_r_*, and *m_data_* are the lengths of the message transmitted in bits, the message received in bits, a sensing data packet in bits, respectively. In addition, *t_id_* is the time period of the idle state and *P* is the dealing rate of the message sensed by sensors. Thus, we have the idle time as

(13) $$t_{id}=t-t_{t}-t_{r}=t-\frac{m_{t}}{Pm_{\text{data}}}-\frac{m_{r}}{Pm_{\text{data}}},$$

where *t* is the data gathering cycle, *t_t_* is the message transmission time, and *t_r_* is the message reception time. Hence, the total energy consumed in a data gathering cycle for a sensor is

(14) $$\begin{array}{ll} E & =\left(\beta_{1}+\beta_{2}d^{\alpha }\right)m_{t}+\beta_{3}m_{r}+\beta_{4}t_{id}Pm_{\text{data}}\\ & =\left[\left(\beta_{1}+\beta_{2}d^{\alpha }\right)-\beta_{4}\right]m_{t}+\left(\beta_{3}+\beta_{4}\right)m_{r}+\beta_{4}tPm_{\text{data}}. \end{array}$$

### 5.1. Scenario I: Without Retransmission

From Equation (13), the energy consumption model for a sensor without retransmission can be expressed as

(15) $$\begin{array}{ll} E_{\text{non}}= & \left[\left(\beta_{1}+\beta_{2}d^{\alpha }\right)-\beta_{4}\right]N_{t,non}m_{\text{data}}\\ & +\left(\beta_{3}-\beta_{4}\right)N_{r,non}m_{\text{data}}+\beta_{4}tPm_{\text{data}}, \end{array}$$

where *N_t_*_,_*_non_* and *N_r_*_,_*_non_* are the numbers of packets transmitted and received without retransmission, respectively.

### 5.2. Scenario II: With Retransmission

When the retransmission is considered, the associated energy consumption for a sensor is

(16) $$\begin{array}{ll} E_{re}= & \left[\left(\beta_{1}+\beta_{2}d^{\alpha }\right)-\beta_{4}\right]\left(N_{t,re,\text{data}}m_{\text{data}}+N_{t,re,\text{ack}}m_{\text{ack}}\right)\\ & +\left(\beta_{3}-\beta_{4}\right)\left(N_{r,re,\text{data}}m_{\text{data}}+N_{r,re,\text{ack}}m_{\text{ack}}\right)+\beta_{4}tPm_{\text{data}}, \end{array}$$

where *m_ack_* is the length of ACK in bits, *N_t_*_,_*_re_*_,_*_data_* and *N_r_*_,_*_re_*_,_*_data_* are the numbers of DATA packets transmitted and received with retransmission, and *N_t_*_,_*_re_*_,_*_ack_* and *N_r_*_,_*_re_*_,_*_ack_* are those of ACK packets, respectively.

## 6. Data Transmission

During the data gathering period, sensors in the WSN collect their surrounding information and send them to the base station hop-by-hop. In this section, to calculate the energy consumed by transmitting and receiving, the possible data transmission paths and their probabilities are analyzed according to the network topology and the routing protocol mentioned in Section 3, and the expected amount of data transmitted and received at each sensor location is derived. Moreover, the network success transmission rate, one constraint in our optimization models in Section 4, is computed as well.

### 6.1. Data Transmission Path

#### Theorem 1

In Figure 1, if $d\leq R_{t}<\sqrt{3}d$, the transmitter can only transmit the sensed DATA to its neighboring sensors centered at the surrounding grids, and the DATA are transmitted from outside to inside layer-by-layer.

**Proof 1** (Proof of Theorem 1).

**Figure 3.**
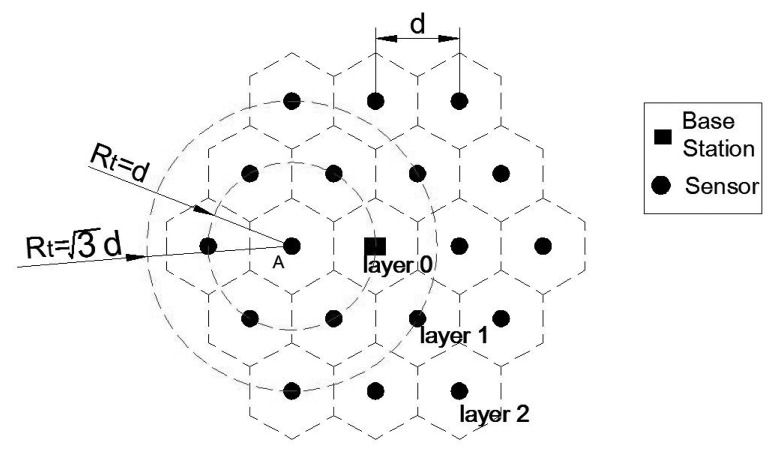
Data transmission analysis.

As shown in Figure 3 (part of Figure 1), Sensor A can transmit its data to other sensors only if the sensor transmission range exceeds the distance between neighboring sensors, *i.e., d* ≤ *R_t_* (see Equation (2)). As mentioned in Equation (3), to guarantee that data collected or received by Sensor A can only be transmitted to its neighbors, $R_{t}<\sqrt{3}d$ should hold to make non-neighboring sensors out of its transmission range, since the distance from Sensor A to its second closest sensor is $\sqrt{3}d$. According to the GPSR protocol, since the sensors in the inner layer are closer to the base station, the transmitter can only send the sensed DATA to an inner layer that is next to it.

Hence, when $d\leq R_{t}<\sqrt{3}d$ holds, the sensor in layer *i* can only receive the data from the nearest sensor in layer (*i* + 1) and send the data to the nearest node in layer (*i* − 1). The assumption that data are transmitted from the outside to the inside, layer-by-layer, is widely used in WSN lifetime or energy optimization problems. Readers are referred to [8,22,23,25,39,43], *etc.*

**Figure 4.**
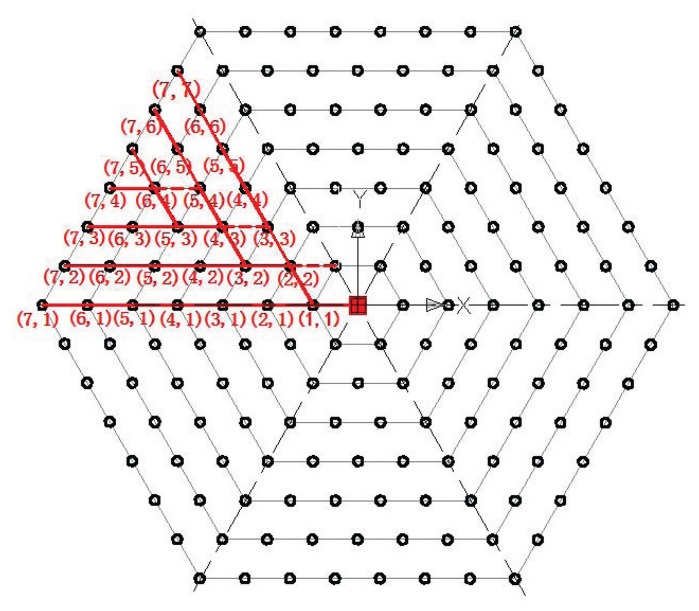
Data transmission path.

Because of the symmetry of a regular hexagon, we only need to study one-sixth of the whole network, *i.e.*, a regular triangular area, to obtain the data transmission path to the base station (expressed by red lines in Figure 4). In the figure, the circles identify the sensor deployment locations. The position of a sensor is numbered by (*i, j*), which indicates that the sensor is located at the *j* − th position of the *i* − th layer. After obtaining data transmission paths for all sensor locations in one regular triangular area, since the data transmission paths for the other five triangles are the same as the one studied, the amount of data transmitted and received at each sensor location in the target area can then be calculated.

Theorem 1 shows that the sensed data will be transmitted to the neighboring sensors in an inner layer. Then, we can find the data transmission path of WSN by computing the distance between neighboring sensors and the base station. For sensors located at (2*x, x* + 1) (*x* = 1, 2, …, e.g., (2,2), (4,3), (6,4), … ), from a vertical line of the triangle, there are two next-hop possibilities since two reception sensor locations in the next inner layer are sharing the same distance from the base station. We assume that if a sensor has two transmission possibilities, the chance of choosing either path is 0.5. For other sensors, there is only one possible next hop, as we can only find one sensor location close to the base station in the next inner layer.

### 6.2. Number of Packets Transmitted or Received

#### 6.2.1. Scenario I: Without Retransmission

Recall that in Section 3, we assume there are *L* layers in the WSN, and, in a data gathering cycle, *a* packets each with *m* bits are sensed and transmitted by one sensor in a single location. However, in each data gathering cycle, the numbers of packets received or transmitted by different sensors may be different. According to the possible data transmission paths obtained in Figure 4, the possible transmitters of each sensor in the outer layer and the corresponding possible receivers in the neighboring inner layer are determined. In Figure 4, one sensor location may have one or two possible transmitters and receivers. Sensor locations with two possible transmitters are the closest sensor locations to the base station in the neighboring inner layer. Locations with two possible receivers are those ones on a vertical line of the triangle as mentioned in Section 6.1. Therefore, three types of locations should be distinguished:

- Location with two possible transmitters and two possible receivers, *i.e.*, (1,1) and (2*x, x* + 1) (*x* = 1, 2, 3, …);
- Location with two possible transmitters and one possible receiver, *i.e.*, (2*x* + 1, *x* + 1) and (2*x* + 1, *x* + 2) (*x* = 1, 2, 3, …);
- Location with one possible transmitter and one possible receiver.

The numbers of packets transmitted and received are calculated as

(17) $$\begin{array}{ccc} N_{t,\text{non}}=\{\begin{array}{ll} \frac{\left(L-i+1\right)\left(L-i+2\right)}{2}a, & \text{Case}\;1,\\ \frac{\left(L-i+1\right)\left(L-i+4\right)}{4}a, & \text{Case}\;2,\\ \left(L-i+1\right)a, & \text{Case}\;3, \end{array} & \text{and} & N_{r,\text{non}}=\{\begin{array}{ll} \frac{\left(L-i\right)\left(L-i+3\right)}{2}a, & \text{Case}\;1,\\ \frac{\left(L-i\right)\left(L-i+5\right)}{4}a, & \text{Case}\;2,\\ \left(L-i\right)a, & \text{Case}\;3, \end{array} \end{array}$$

respectively (*N_r_*_,_*_non_* = *N_t_*_,_*_non_* − 1).

#### 6.2.2. Scenario II: With Retransmission

Under the assumptions in Section 3.2, simplified CSMA/CA protocol without RTS/CTS is adopted to improve the success rate of data transmission. There are three situations in a data transmission process: (1) the receiver fails to receive the DATA; (2) the receiver successfully receives the DATA but the transmitter fails to receive the ACK; and (3) both DATA and ACK are successfully received. For the first two situations, the transmitter will retransmit the DATA until it receives the ACK message from the receiver or the maximum number of retry attempts is reached.

Assume each transmission is an independent event. Let *A* and *B* be the retransmission rates of DATA and ACK, respectively. All the transmission possibilities and the corresponding probabilities in one hop are enumerated in Table 1 (the maximum number of retry attempts is 2). Take the case in the first row as an example, it describes the situation that: (1) the transmitter sends DATA to the receiver; (2) the receiver successfully receives it then sends an ACK back to the transmitter; and (3) the receiver again successfully receives the ACK and no retransmission occurs. The transmission process is denoted in Columns 1–6, where 1, 0 and N/A represent the transmission success, failure, and no transmission, respectively. In this case, the number of retry attempts for either DATA or ACK is 1, and this information is recorded in Columns 7 and 8. Since packet transmission is an independent event, the probability that this situation happens can be calculated via the multiplication rule, and the calculation result, (1 − *A*)(1 − *B*), is in Column 9. When DATA is finally successfully transmitted to the receiver, Column 10 shows “S”. In Column 10, S and F denote whether or not the sensed DATA can be transmitted successfully within the maximum number of retry attempts.

Since packet transmission is an independent event, the probabilities of transmitting the DATA and the ACK *n* times can be written as

(18) $$\omega_{\text{data},n}=\{\begin{array}{ll} \left(1-A\right)\left(1-B\right), & n=1,\\ A\left(1-A\right)\left(1-B\right)+B{\left(1-A\right)}^{2}\left(1-B\right), & n=2,\\ A^{2}+2AB\left(1-A\right)+B^{2}{\left(1-A\right)}^{2}, & n=3, \end{array}$$

and

(19) $$\omega_{\text{ack},n}=\{\begin{array}{ll} \left(1-A^{2}\right)\left(1-B\right)+A^{2}\left(1-A\right)\left(1+2B\right), & n=1,\\ B{\left(1-A\right)}^{2}\left(1-B\right)+AB{\left(1-A\right)}^{2}\left(2+B\right), & n=2,\\ B^{2}{\left(1-A\right)}^{3}, & n=3, \end{array}$$

respectively. Thus, the expected numbers of transmission attempts of DATA and ACK, respectively, are

(20) $$\{\begin{array}{l} \omega_{\text{data}}=\omega_{\text{data},1}+2\omega_{\text{data},2}+3\omega_{\text{data},3},\\ \omega_{\text{ack}}=\omega_{\text{ack},1}+2\omega_{\text{ack},2}+3\omega_{\text{ack},3}. \end{array}$$

Hence, the numbers of packets transmitted and received, for both DATA and ACK, under the retransmission can be expressed as

(21) $$N_{t,re,\text{data}}=\{\begin{array}{ll} \frac{\left(L-i+1\right)\left(L-i+2\right)}{2}a\omega_{\text{data}}, & \text{Case}1,\\ \frac{\left(L-i+1\right)\left(L-i+4\right)}{4}a\omega_{\text{data}}, & \text{Case}2,\\ \left(L-i+1\right)a\omega_{\text{data}}, & \text{Case}3, \end{array}$$

(22) $$N_{t,re,\text{ack}}=\{\begin{array}{ll} \frac{\left(L-i\right)\left(L-i+3\right)}{2}a\omega_{\text{ack}}, & \text{Case}1,\\ \frac{\left(L-i\right)\left(L-i+5\right)}{4}a\omega_{\text{ack}}, & \text{Case}2,\\ \left(L-i\right)a\omega_{\text{ack}}, & \text{Case}3, \end{array}$$

and

(23) $$N_{r,re,\text{data}}=\{\begin{array}{ll} \frac{\left(L-i\right)\left(L-i+3\right)}{2}a\omega_{\text{data}}, & \text{Case}1,\\ \frac{\left(L-i\right)\left(L-i+5\right)}{4}a\omega_{\text{data}}, & \text{Case}2,\\ \left(L-i\right)a\omega_{\text{data}}, & \text{Case}3, \end{array}$$

(24) $$N_{r,re,\text{ack}}=\{\begin{array}{ll} \frac{\left(L-i+1\right)\left(L-i+2\right)}{2}a\omega_{\text{ack}}, & \text{Case}1,\\ \frac{\left(L-i+1\right)\left(L-i+4\right)}{4}a\omega_{\text{ack}}, & \text{Case}2,\\ \left(L-i+1\right)a\omega_{\text{ack}}, & \text{Case}3, \end{array}$$

respectively.

### 6.3. Success Rate of Data Transmission

Assume at least one sensor is functioning at each position, the success rate of data transmission for the entire WSN is

(25) $$S=\prod_{i=1}^{L}\prod_{j=1}^{L}S{\left(i,j\right)}^{N_{t}\left(i,j\right)},$$

where *S*(*i, j*) is the success rate of data transmission and *N_t_*(*i, j*) is the number of packets transmitted at position (*i, j*). If no retransmission occurs then *S*(*i, j*) = *S_non_*(*i, j*). Otherwise, *S*(*i, j*) = *S_re_*(*i, j*).

#### 6.3.1. Scenario I: Without Retransmission

The success rate of data transmission can be calculated according to the results in Column 10 of Table 1. If retransmission is not considered, the data will only be transmitted once. The rate of successfully sending a packet from a sensor in grid (*i, j*) to the next hop can be expressed as

(26) $$S_{\text{non}}\left(i,j\right)=1-A.$$

#### 6.3.2. Scenario II: With Retransmission

When retransmission is considered, by summing all of the successful transmission probabilities in Table 1, the success rate of data transmission in one hop can be written as

(27) $$S_{re}\left(i,j\right)=1-A^{3}-2A^{2}B\left(1-A\right)-AB^{2}{\left(1-A\right)}^{2}.$$

## 7. Case Study

We conduct case studies for a WSN whose parameters are presented in Table 2. For deployment-based lifetime optimization, Section 7.1 compares the result of the case with retransmission with that of the case without retransmission. The results verify the importance of considering retransmission. Ignoring retransmission may overestimate the lifetime and yield a non-optimal deployment. Section 7.2 analyzes the optimal uniform and non-uniform deployments. The results show that the non-uniform deployment can provide longer lifetime with a given number of sensors, and more sensors can be saved for a long-lifetime requirement. The larger the monitoring area is, the more noticeable the strength of non-uniform deployment will become. Compared with the random deployed WSN, the advantages of our hexagonal deployment are verified in Section 7.3.

### 7.1. Importance of Considering Retransmission

Either with or without retransmission, the optimal distance between the neighboring sensors can be obtained from the associated optimization model. The results of optimal lifetime are shown in Tables 3 and 4 for uniform and non-uniform deployments, respectively.

From Tables 3 and 4, one can see that the optimal sensor deployment scheme of WSN with retransmission (WSNWR) is quite different from that of WSN without retransmission (WSNWoR). Basically, the distance between sensors for WSNWR is longer than that of WSNWoR, which results in a smaller number of layers. From the two tables above, one can see that the success transmission rate is much higher with retransmission, as retransmission can improve the success transmission rate between hops, which enhances the whole WSN. The lifetime of WSNWoR is overestimated since the energy consumption of retransmission is not included. Moreover, to satisfy a given *τ* = 365 days, the total number of sensors required for WSNWR is less than that of WSNWoR. This is because the WSN will be more reliable when retransmission is considered. Therefore, it is meaningful to consider retransmission in the sensor-deployment optimization for a WSN. In the following sections, we only focus on the WSN under retransmission.

### 7.2. Comparison of Uniform and Non-Uniform Deployment

Tables 3 and 4 also show that the optimal sensor deployment schemes are the same in uniform and non-uniform distributions, since the optimal sensor distance is the one that can consume the least energy with one sensor located in the center of each grid. In addition, under the optimal sensor deployment, *d* = 58.97 m and *L* = 9, the total number of sensors required to survive for 365 days in non-uniform deployment is 3006, whereas the number is 3510 in uniform deployment. Moreover, when the numbers of sensors deployed are the same (*N* = 3510), the lifetime of WSN is 365 days if sensors are uniformly deployed, whereas the maximum lifetime can extend to 418 days under the optimal non-uniform deployment via Equation (11). All of the above results imply that the non-uniform sensor deployment scheme provides longer lifetime than that of the uniform one.

**Figure 5.**
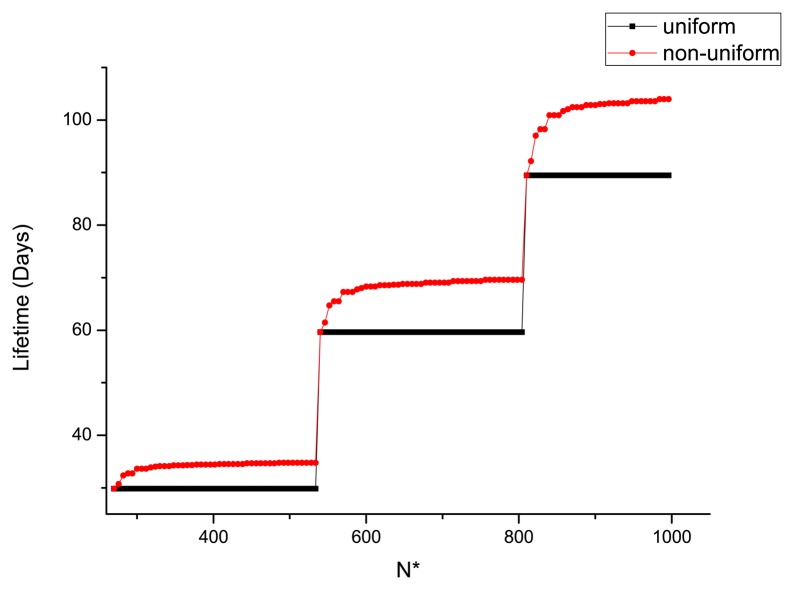
Optimal lifetime under different N*.

Particularly, the constraints of the maximum number of sensors, the required lifetime and the radius of the circular target area will affect the optimization results. When the constraint N* (the maximum number of sensors) varies, the optimal lifetime of WSN is illustrated in Figure 5. One can see that the non-uniform deployment is useful in prolonging the lifetime of WSN. As the numbers of packets transmitted and received at some locations are the same (see Figure 4), the energy consumptions for sensors in these locations are the same. Thus, one can see from Figure 5 that the optimal lifetime for a certain number of sensors does not change continuously.

**Figure 6.**
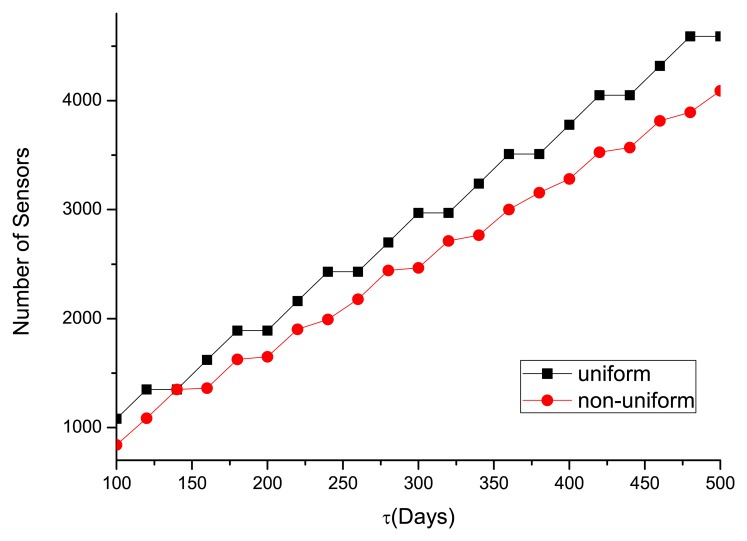
Optimal numbers of sensors deployed under different τ*.

**Figure 7.**
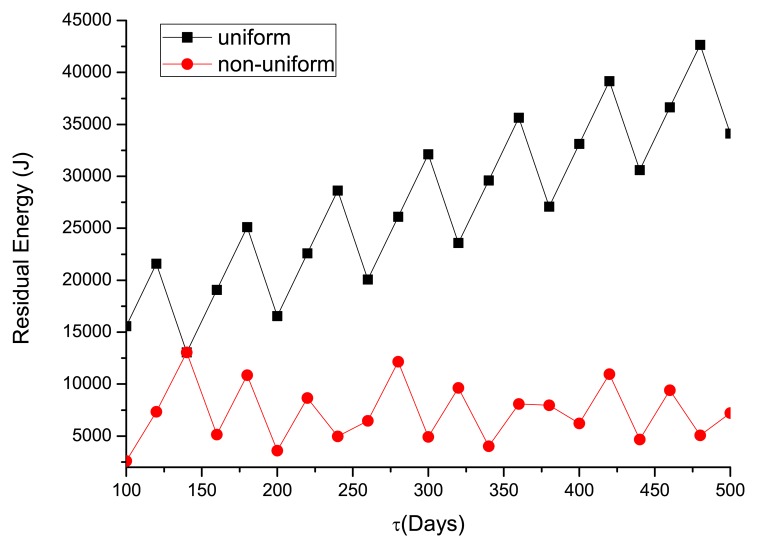
Residual energy in WSN under different τ*.

To satisfy a certain required lifetime (τ*) varying from 100 to 500 days, the optimal number of sensors deployed in the WSN is shown in Figure 6, and the associated energy left when the life ends is illustrated in Figure 7. One can see that more sensors are needed to satisfy the long-lifetime requirement. Compared with the uniform deployment, the saving in sensors by the non-uniform WSN is increasingly evident at longer required lifetime. In Figure 7, the residual energy for the non-uniform deployment is much less than the uniform one. As the optimal lifetime for a certain number of sensors does not change continuously (see Figure 5), the residual energy is not always monotonically increasing in the required lifetime.

**Figure 8.**
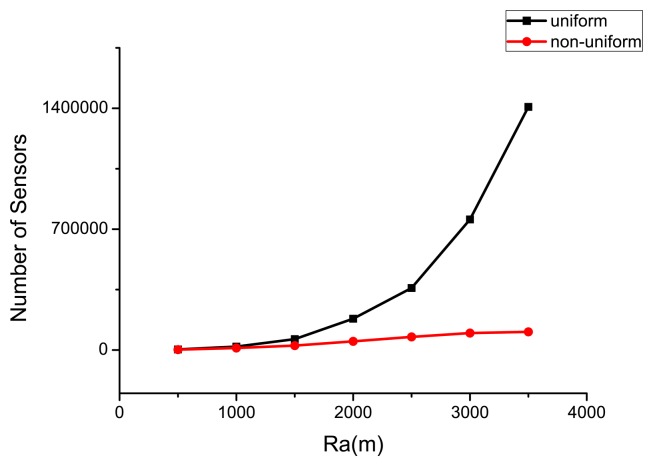
Optimal number of sensors deployed under different *R_a_*.

**Figure 9.**
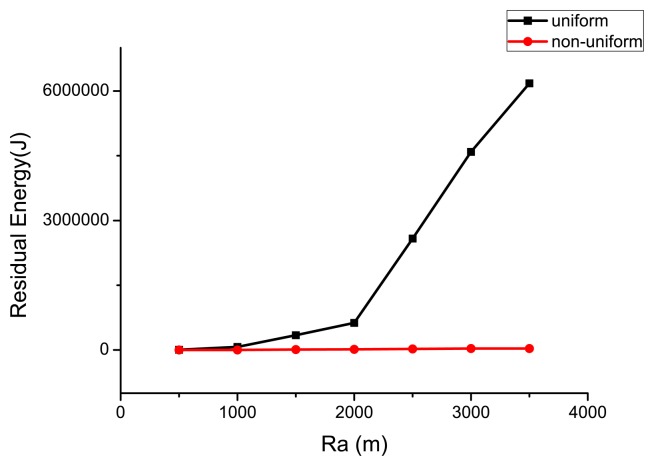
Residual energy in WSN under different *R_a_*.

Similarly, when the radius of the circular target area *R_a_* varies, the optimal number of sensors deployed (compare the uniform case with the non-uniform case) in the WSN is shown in Figure 8 (for a lifetime of 365 days). The associated residual energy when the life ends (under the optimal sensor deployment scheme) is illustrated in Figure 9. Due to the energy hole problem, sensors that are close to the base station need to transmit more sensing data, and they consume energy much faster than the ones in the periphery of the target area. For uniformly deployed WSN, the unbalanced energy consumption cannot be avoided, and energy will be surely left. The non-uniform deployment scheme can solve this problem, and both the number of sensors needed and residual energy of the non-uniform deployment are much less than that of the uniform one. In fact, the number of sensors and the residual energy are relatively stable as *R_a_* increases under the non-uniform scheme, whereas they increase sharply for the uniform one. This implies that the non-uniform scheme is much better than the uniform one when *R_a_* becomes large.

### 7.3. Effectiveness of the Proposed Sensor Deployment Scheme

To verify the effectiveness of our sensor deployment scheme, we use the Monte Carlo method to simulate the lifetime of the WSN under a random sensor deployment scheme in order to provide comparisons. The lifetime of a hexagonal deployed WSN is also simulated to analyze the simulation error. Our Monte Carlo simulation was implemented in MATLAB, and the general simulation procedure is described as follows.

- Step 1. Generate the sensor locations according to the deployment scheme (*i.e.*, the random topology or the hexagonal topology);
- Step 2. Assign the corresponding attributes to each sensor, including initial energy, packet length of sensing data, number of packet sensed in a data gathering cycle, sensing range, transmission range, *etc.*;
- Step 3. Execute the following steps at each data gathering time until the WSN life ends;
  I Determine the active sensors according to the sleep/wake-up protocol (*i.e.*, PEAS protocol)
  II Generate sensing data at all alive sensors,
  III Transmit the sensing data to the base station hop-by-hop based on the routing protocol (*i.e.*, GPSR protocol). For each DATA packet transmission between two hops, implement the following procedures,
    – (i) Transmit the DATA to its receiver, and determine whether it is received successfully according to the DATA retransmission rate;
    – (ii) If the DATA is received by the receiver, transmit an ACK back to the transmitter and determine whether the ACK is received successfully according to the ACK retransmission rate, else go to step (iii);
    – (iii) If the transmitter fails to receive the ACK and the maximum number of retry times is not achieved, go to step (i) to retransmit the DATA, else go to step (iv);
    – (iv) Calculate the energy consumption of each DATA or ACK transmission and reception. If the energy of a sensor is exhausted, remove it from the WSN.
  IV Evaluate the coverage rate using pixel method proposed in [44]. If the coverage rate cannot satisfy the coverage requirement, terminate the simulation, and record the time as the lifetime for this simulation.
- Step 4. Count the number of DATA that is successfully received by the base station, and evaluate the success transmission rate;
- Step 5. Go to Step 1 until the number of simulation runs is achieved. Otherwise, go to Step 6;
- Step 6. Calculate the average lifetime and success transmission rate.

#### 7.3.1. Simulation Error Analysis

To verify the reliability of our simulation, the lifetime of a WSN under our hexagonal deployment scheme is simulated with only one sensor per grid to explain the associated error of our Monte Carlo method. The simulation parameters are taken from Table 2 and the optimization results of uniform sensor deployment under retransmission are shown in Table 3, where *d* = 58.97 m and *L* = 9. The simulation is repeated for 1000 times. The error of lifetimes of the analytical solution and the simulation result is 5.5 s, and the error of the corresponding success transmission rates is 0.0000117.

#### 7.3.2. Comparison of Hexagonal and Random Sensor Deployments

Our optimal hexagonal deployment scheme and the random one are compared under retransmission via simulation. The simulation parameters are the same as in Section 7.3.1. As *L* = 9, the number of sensors in the hexagonal deployed WSN is *N* = 270. In the WSN under the random deployment scheme, the only difference is that 270 sensors are randomly deployed in the target area.

In the comparison simulation, the WSN lifetime is defined as the time period between the starting time and the time when the coverage falls below 85%. We do not consider 100% coverage since it is calculated based on the pixel method, and there is some pixel error in the coverage rate calculation. The simulation is repeated for 1000 times, and the results are presented in Table 5.

From Table 5, one can see that the lifetime and the success transmission rate of hexagonal deployed WSN are 1.022 and 3.012 times of that of random deployed WSN, respectively. Apparently, the lifetime of the sensor deployment scheme proposed in this work is better than that of a random deployment and gives a much higher success transmission rate.

#### 7.3.3. Case with Redundancy

**Figure 10.**
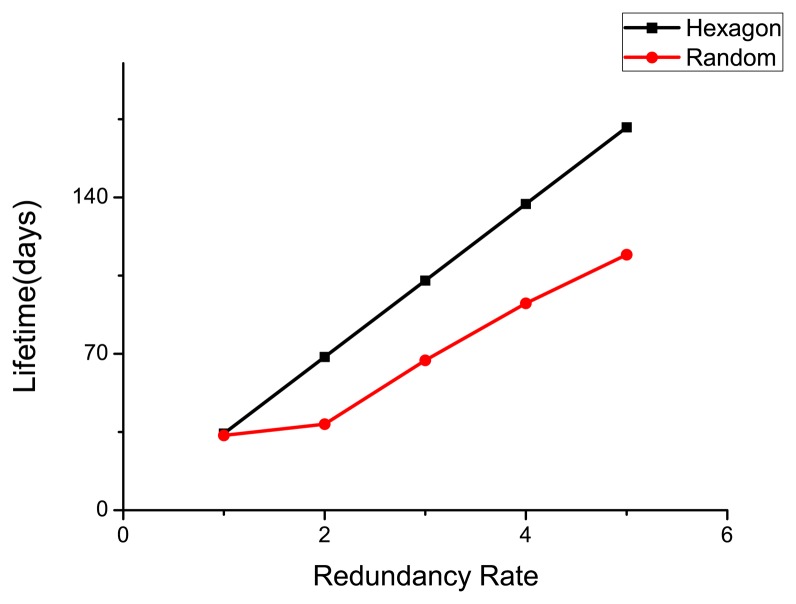
Lifetime under different redundancy rate.

**Figure 11.**
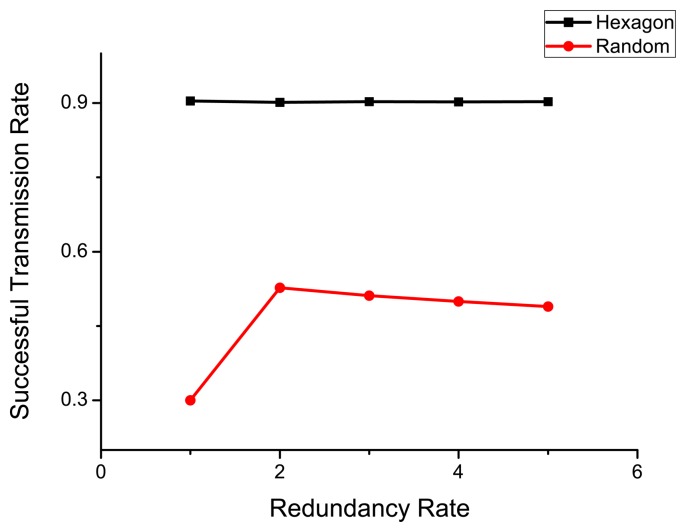
Data successful transmission rate (S) under different redundancy.

Since it is easy to run out of energy for a single sensor, it is common to deploy redundancy in each grid. As the sleep/wake-up protocol PEAS is adopted in our model, only one sensor will be active per grid. We compare the lifetime and success transmission rate considering the number of redundant units as 1, 2, 3, 4, 5, respectively. The simulation results are shown in Figures 10 and 11.

As shown in Figure 9, the lifetimes of both deployment models increase as the redundancy increases. The lifetime of hexagonal deployment is always longer than that of a random one, and the difference between them grows as more redundant units become available. The data success transmission rate of hexagonal deployment is also higher than that of the random one. Figure 10 implies that when the redundancy rate is higher than 2, the value of *S* does not change significantly. However, for the random deployment, it increases significantly as the number jumps from 1 to 2 since the sensor density is too low when the redundancy is 1.

Therefore, we can conclude that redundancy is useful in improving the lifetime of WSN under the constraint of a required success transmission rate. Moreover, the hexagonal deployment that we proposed is much better than a random one under different redundancy rates.

## 8. Conclusions

In this paper, a homogeneous WSN deployment scheme based on the regular hexagonal topology is analyzed. To maximize the WSN lifetime, both uniform and non-uniform deployment optimization models are proposed with considerations of coverage, connectivity and success transmission rate. To ensure the survival of the WSN for a given lifetime, the method of obtaining the minimal total number of sensors needed is also presented. The energy consumption and the data transmission path of each sensor are quantified under retransmission, which makes our work more realistic. Our case studies verify that it is necessary to consider retransmission in the deployment of WSN; otherwise, the lifetime will be overestimated. The optimal lifetime of the non-uniform sensor deployment is longer than that of the uniform one, and, particularly in large target areas, the total number of sensors required is much less. In addition, simulation results show that the optimal sensor deployment scheme obtained by the optimization models always has a longer lifetime and a higher data success transmission rate than a random scheme.

This paper focuses on homogeneous structured WSN with a circular target area and a base station in the center. For other types of WSNs, such as heterogeneous structured WSN with other monitoring target (e.g., a rectangular or irregular area and a base station in the middle or on the edge), unstructured random WSN, heterogeneous WSN, *etc.*, retransmission should be considered in the deployment-based lifetime optimization as well. The method of integrating retransmission into energy consumption proposed herein can still be applied, but the optimization model and the data transmission path should be modified to adapt to the specific problem and new deployment scheme may be proposed. We will study these types of WSNs in our future research.

## Figures and Tables

**Table 1.** Data transmission possibilities in a certain hop.

**Transmission Results**						**Number of Attempts**		**Probability**	**Success or Failure**

**Attempt 1**		**Attempt 2**		**Attempt 3**					

**DATA**	**ACK**	**DATA**	**ACK**	**DATA**	**ACK**	**DATA**	**ACK**		
1	1	N/A	N/A	N/A	N/A	1	1	(1 − *A*)(1 − *B*)	S
1	0	1	1	N/A	N/A	2	2	*B*(1 − *A*)^2^(1 − *B*)	S
0	N/A	1	1	N/A	N/A	2	1	*A*(1 − *A*)(1 − *B*)	S
1	0	1	0	1	1	3	3	*B*^2^(1 − *A*)^3^(1 − *B*)	S
1	0	1	0	1	0	3	3	*B*^3^(1 − *A*)^3^	S
1	0	10	0	0	N/A	3	2	*AB*^2^(1 − *A*)^2^	F
1	0	0	N/A	1	1	3	2	*AB*(1 − *A*)^2^(1 − *B*)	S
1	0	0	N/A	1	0	3	2	*AB*^2^(1 − *A*)^2^	S
1	0	0	N/A	0	N/A	3	1	*A*^2^*B*(1 − *A*)	F
0	N/A	1	0	1	1	3	2	*AB*(1 − *A*)^2^(1 − *B*)	S
0	N/A	1	0	1	0	3	2	*AB*^2^(1 − *A*)^2^	S
0	N/A	1	0	0	N/A	3	1	*A*^2^*B*(1 − *A*)	F
0	N/A	0	N/A	1	1	3	1	*A*^2^(1 − *A*)(1 − *B*)	S
0	N/A	0	N/A	1	0	3	1	*A*^2^*B*(1 − *A*)	S
0	N/A	0	N/A	0	N/A	3	0	*A*^3^	F

**Table 2.** Parameters of the homogeneous WSN. The details of the symbols can be seen in the Appendix.

**Parameter**	**Value**	**Parameter**	**Value**	**Parameter**	**Value**
*α*	4	*E*_0_	54 J	*P*	1 packet/s
*β*_1_	5.0 × 10^−8^ J/bit	*f*	8.68 × 10^8^ Hz	*P_t_*	−6 dB
*β*_2_	1.3 × 10^−15^ J/bit	*l_ack_*	120 bits	*R_s_*	50 m
*β*_3_	5.0 × 10^−8^ J/bit	*l_data_*	160 bits	*R_t_*	80 m
*β*_4_	4.0 × 10^−8^ J/bit	*m_ack_*	120 bits	*S_r_*	−98 dB
*R_a_*	500 m	*m_data_*	448 bits	*t*	300 s
S*	0.6	*a*	1	τ*	365 days

**Table 3.** Optimization results of uniform deployment. The details of the symbols can be seen in the Appendix.

**Optimal Solutions**	**With Retransmission**	**Without Retransmission**
*d*	58.97 m	50.00 m
*L*	9	11
*S*	0.99991	0.61510
*τ*	715.80 h	725.44 h
*N*	3510	5148

Note: the optimal lifetime is obtained by deploying only one sensor in each regular hexagonal grid using Equation (7), and the minimal *N* is obtained using Equation (9).

**Table 4.** Optimization results of non-uniform deployment. The details of the symbols can be seen in the Appendix.

**Optimal Solutions**	**With Retransmission**	**Without Retransmission**
*d*	58.97 m	50.00 m
*L*	9	11
*S*	0.99991	0.61510
*τ*	835.06 h	822.32 h
*N*	3006	4392

Note: the optimal lifetime is obtained by the optimization model in Equation (10) under the constraint of N* = 500, and the minimal *N* is obtained using Equation (11).

**Table 5.** Optimization results of uniform deployment.

**Simulation Results**	**Hexagonal Deployment**	**Random Deployment**
*τ*	822.48 h	804.72 h
*S*	0.903943	0.300116

## Appendix

### A. Acronyms

**Table:**

ACK	Acknowledgement
CSMA/CA	Carrier Sense Multiple Access/Collision Avoidance
DATA	Sensing Data
GPSR	Greedy Perimeter Stateless Routing
PEAS	Probing Environment and Adaptive Sleeping
RTS/CTS	Request to Send/Clear to Send
WSN	Wireless Sensor Network
WSNWR	Wireless Sensor Network with Retransmission
WSNWoR	Wireless Sensor Network without Retransmission

### B. Symbols

**Table:**

*α*	the path loss exponent
*β*_1_	the energy spent in the electronics circuitry for transmitting each bit data
*β*_2_	the coefficient affected by the transmit amplifier efficiency, antenna gains and other system parameters
*β*_3_	the energy spent in the electronics circuitry for receiving each bit data
*β*_4_	the energy spent for each bit message in the idle state
*τ*	the optimal lifetime
*τ**	the lifetime requirement of the WSN
*ω_ack_*	the expected number of transmission attempts of an ACK packet
*ω_ack_*_,_*_n_*	the probability of transmitting an ACK packet *n* times
*ω_data_*	the expected number of transmission attempts of a DATA packet
*ω_data_*_,_*_n_*	the probability of transmitting a DATA packet *n* times
*a*	the number of packets sensed by each sensor in each data gathering cycle
*A*	the retransmission rate of a DATA packet
*B*	the retransmission rate of an ACK packet
*C*	the speed of light
*d*	the distance between the neighboring sensors
*d_c_*(*i*)	the circumcircle radius of a sensor in layer *i*
*E*	the total energy consumed by a sensor in a data gathering cycle
*E*_0_	the initial energy of a sensor
*E_i_*_,_*_j_*	the energy consumption at location (*i, j*) in a data gathering cycle
$E_{i,j}^{\prime }$	the optimal energy consumed by the sensor at location (*i, j*) in a data gathering cycle
*E_id_*	the energy consumption in the idle state
*E_non_*	the energy consumption of a sensor without retransmission in a data gathering cycle
*E_r_*	the energy consumption of reception
*E_re_*	the energy consumption of a sensor with retransmission in a data gathering cycle
*E_t_*	the energy consumption of transmission
*f*	the bandwidth
*i*	the layer number
*j*	the location number in a layer
*l*	the length of the packet preamble
*L*	the number of layers
*l_ack_*	the preamble length of the ACK packet
*l_data_*	the preamble length of the DATA packet
*m*	the length of a single packet
*m_ack_*	the length of the ACK packet
*m_data_*	the length of the DATA packet
*m_r_*	the length of the message received
*m_t_*	the length of the message transmitted
*n*	the transmission times
*n_i_*_,_*_j_*	the number of sensors deployed at location (*i, j*)
*N*	the optimal total number of sensors deployed in the target area
*N**	the maximum number of sensors allowed to be deployed in the target area
*N_t_*(*i,j*)	the number of packets transmitted at location (*i, j*)
*N_t_*_,_*_non_*	the numbers of packets transmitted without retransmission
*N_r_*_,_*_non_*	the numbers of packets received without retransmission
*N_t_*_,_*_re_*_,_*_ack_*	the numbers of ACK packets transmitted with retransmission
*N_r_*_,_*_re_*_,_*_ack_*	the numbers of ACK packets received with retransmission
*N_t_*_,_*_re_*_,_*_data_*	the numbers of DATA packets transmitted with retransmission
*N_r_*_,_*_re_*_,_*_data_*	the numbers of DATA packets received with retransmission
*P*	the dealing rate of the message sensed by sensors
*P_t_*	the transmission power
*R_a_*	the radius of the circular target area
*R_s_*	the sensing radius of sensors
*R_t_*	the transmission radius of sensors
*RR*(*d*)	the retransmission rate
*S*	the success transmission rate
*S**	the requirement of the success transmission rate
*S*(*i, j*)	the success transmission rate at location (*i, j*)
*S_non_*(*i, j*)	the success transmission rate without retransmission at location (*i, j*)
*S_r_*	the receiver's sensitivity
*S_re_*(*i, j*)	the success transmission rate with retransmission at location (*i, j*)
*t*	the data gathering cycle
*t_id_*	the time period of the idle state
*t_r_*	the message reception time
*t_t_*	the message transmission time

## References

[1] Jafari R., Encarnacao A., Zahoory A., Dabiri F., Noshadi H., Sarrafzadeh M. Wireless sensor networks for health monitoring.

[2] Bokareva T., Hu W., Kanhere S., Ristic B., Gordon N., Bessell T., Rutten M., Jha S. Wireless sensor networks for battlefield surveillance.

[3] Garcia-Sanchez A.-J., Garcia-Sanchez F., Losilla F., Kulakowski P., Garcia-Haro J., Rodríguez A., López-Bao J.-V., Palomares F. (2010). Wireless sensor network deployment for monitoring wildlife passages. Sensors.

[4] Yoo S.E. (2013). A wireless sensor network-Based portable vehicle detector evaluation system. Sensors.

[5] Wu F.J., Kao Y.F., Tseng Y.C. (2011). From wireless sensor networks towards cyber physical systems. Pervasive Mob. Comput..

[6] Xu K., Wang Q., Hassanein H., Takahara G. Optimal wireless sensor networks (WSNs) deployment: minimum cost with lifetime constraint.

[7] Asorey-Cacheda R., García-Sánchez A.J., García-Sánchez F., García-Haro J., González-Castano F.J. (2013). On maximizing the lifetime of wireless sensor networks by optimally assigning energy supplies. Sensors.

[8] Wu X., Chen G., Das S.K. (2008). Avoiding energy holes in wireless sensor networks with nonuniform node distribution. IEEE Trans. Parallel Distrib. Syst..

[9] Anastasi G., Conti M., Francesco M.D., Passarella A. (2009). Energy conservation in wireless sensor networks: A survey. Ad Hoc Netw..

[10] Younis M., Akkaya K. (2008). Strategies and techniques for node placement in wireless sensor networks: A survey. Ad Hoc Netw..

[11] Silva I., Guedes L.A., Portugal P., Vasques F. (2012). Reliability and availability evaluation of wireless sensor networks for industrial applications. Sensors.

[12] Rosberg Z., Liu R.P., Dinh T.L., Dong Y., Jha S. (2010). Statistical reliability for energy efficient data transport in wireless sensor networks. Wirel. Netw..

[13] Wen H., Lin C., Ren F., Yue Y., Huang X. Retransmission or redundancy: transmission reliability in wireless sensor networks.

[14] She H., Lu Z., Jantsch A., Zhou D., Zheng L.R. Analytical evaluation of retransmission schemes in wireless sensor networks.

[15] Wen H., Lin C., Ren F., Yang H., He T., Dutkiewicz E. Joint adaptive redundancy and partial retransmission for reliable transmission in wireless sensor networks.

[16] Onur E., Ersoy C., Deliç H., Akarun L. (2007). Surveillance wireless sensor networks: Deployment quality analysis. IEEE Netw..

[17] Ceriotti M., Mottola L., Picco G.P., Murphy A.L., ŞtefanGunaă., Corrà M., Pozzi M., Zonta D., Zanon P. Monitoring heritage buildings with wireless sensor networks: The torre aquila deployment.

[18] Gallart V., Felici-Castell S., Delamo M., Foster A., Perez J.J. Evaluation of a real, low cost, urban WSN deployment for accurate environmental monitoring.

[19] Shen Y., Yang P., Zhang P., Luo Y., Mei Y., Cheng H., Jin L., Liang C., Wang Q., Zhong Z. (2013). Development of a multitype wireless sensor network for the large-scale structure of the national stadium in China. Int. J. Distrib. Sens. Netw..

[20] Navarro M., Davis T.W., Liang Y., Liang X. A study of long-term WSN deployment for environmental monitoring.

[21] Liu Z., Li Y., Chen Q., Lin J. Control lifetime by density in wireless sensor networks.

[22] Wang J. The placement scheme for static nodes in a circular WSN area based on the analysis of density and energy.

[23] AbdelSalam H.S., Olariu S. HexNet: hexagon-based localization technique for wireless sensor networks.

[24] Chiang Y.K., Wang N.C., Hsieh C.H. (2014). A cycle-based data aggregation scheme for grid-based wireless sensor networks. Sensors.

[25] Fan T., Teng G., Huo L. (2014). A pre-determined nodes deployment strategy of two-tiered wireless sensor networks based on minimizing cost. Int. J. Wirel. Inf. Netw..

[26] Gupta M., Krishna C.R., Prasad D. SEEDS: Scalable energy efficient deployment scheme for homogeneous wireless sensor network.

[27] Tian H., Shen H. An optimal coverage scheme for wireless sensor network.

[28] Yedavalli R.K., Belapurkar R.K. (2011). Application of wireless sensor networks to aircraft control and health management systems. J. Control Theory Appl..

[29] Leipold F., Tassetto D., Bovelli S. (2013). Wireless in-cabin communication for aircraft infrastructure-A holistic approach for on-board high data-rate UWB network. Telecommun Syst..

[30] Wang H., Hu T. (2014). Application and airworthiness research of airborne wireless sensor networks. Aeronaut. Sci. Technol..

[31] Kapnadak V., Coyle E.J. (2014). Optimal Nonuniform Deployment of Sensors for Distributed Detection in Wireless Sensor Networks. ACM Trans. Sens. Netw..

[32] Landsiedel O., Wehrle K., Gotz S. Accurate prediction of power consumption in sensor networks.

[33] (2009). CC1000-Datasheet. Single-chip very low power RF transceiver (Rev. A)..

[34] Karp B., Kung H.T. GPSR: greedy perimeter stateless routing for wireless networks.

[35] Ye F., Zhong G., Cheng J., Lu S., Zhang L. PEAS: a robust energy conserving protocol for long-lived sensor networks.

[36] Zuniga M., Krishnamachari B. Analyzing the transitional region in low power wireless links.

[37] (2006). Wireless Medium Access Control (MAC) and Physical Layer (PHY) Specifications for Low-Rate Wireless Personal Area Networks (WPANs). IEEE802.15.4.

[38] Dietrich I., Dressler F. (2009). On the lifetime of wireless sensor networks. ACM Trans. Sens. Netw..

[39] Olariu S., Stojmenovic I. Design guidelines for maximizing lifetime and avoiding energy holes in sensor networks with uniform distribution and uniform reporting.

[40] Stemm M., Katz R.H. (1997). Measuring and reducing energy consumption of network interfaces in hand-held devices. IEICE Trans. Commun..

[41] Chen B., Jamieson K., Balakrishnan H., Morris R. (2002). Span: an energy-efficient coordination algorithm for topology maintenance in ad hoc wireless networks. Wirel. Netw..

[42] Gao Q., Blow K.J., Holding D.J., Marshall I.W., Peng X. (2006). Radio range adjustment for energy efficient wireless sensor networks. Ad Hoc Netw..

[43] Song C., Liu M., Cao J., Zheng Y., Gong H., Chen G. (2009). Maximizing network lifetime based on transmission range adjustment in wireless sensor networks. Comput. Commun..

[44] Li R., Sun G., Liao H., Huang N. Lifetime analysis of wireless sensor networks under retransmission.

